# An explorative study to assess the psychosocial impact of infertility on female Infertile Patients

**DOI:** 10.1192/j.eurpsy.2022.1199

**Published:** 2022-09-01

**Authors:** B. Ghosh Dastidar

**Affiliations:** Royal Wolverhampton NHS Trust, University of Birmingham, Psychiatry, WVQP, United Kingdom

**Keywords:** Psychopathology, Depression, Anxiety, Infertility

## Abstract

**Introduction:**

The primary aim of our study was to assess the psychoscial impact of infertility amongst female infertile patients. We selected infertile women visiting the gynaecological OPD of R.G.Kar Medical College and Hospital - one of the busiest and most reputed government hospitals in eastern India. Most of the research on psychological aspects on infertility has been done in the developed rich nations of the world, our study is one of the very few to have been conducted in a developing nation like India.

**Objectives:**

Incidence of depression , psychopathology and anxiety in female infertile patients in comparison to control fertile group. Impairment in quality of life Impact of other variable factors

**Methods:**

Source of data : RGK Medical College, Kolkata, India

Sampling technique : Consecutive Random Sampling

Tools for data collection: Beck’s Depression Inventory, Beck’s Anxiety Inventory, SF36, SCL 90, Mini International Neuropsychiatric Interview, Self Reporting Questionnaire, Socio Demographic Proforma.

Data Analysis: The data was collected and analyzed by means of descriptive and inferential statistics.

Inferential statistics- Data analyzed by using SPSS. The relationship between continuous and binary explanatory variables with SF36, SCL 90, BDI and BAI scores were assessed using unpaired t test.

**Results:**

Statistical analysis by independent t test shows significant increased levels of depression, anxiety, significant difference in psychopathology and quality of life in the 2 study groups.

**Conclusions:**

Infertility has a significant impact on psychosocial well being of infertile patients, Greater collaboration is required between psychiatrists, psychologists and infertility specialists to assist infertile couples.

**Disclosure:**

No significant relationships.

